# *In situ* characterization of the high pressure – high temperature melting curve of platinum

**DOI:** 10.1038/s41598-019-49676-y

**Published:** 2019-09-10

**Authors:** Simone Anzellini, Virginia Monteseguro, Enrico Bandiello, Agnès Dewaele, Leonid Burakovsky, Daniel Errandonea

**Affiliations:** 1 Diamond Light Source Ltd, Diamond House, Harwell Science Campus, Didcot, Oxfordshire OX11 0DE UK; 2 Departamento de Física Aplicada - Instituto de Ciencia de Materiales, Matter at High Pressure (MALTA) Consolider Team, Universidad de Valencia, Edificio de Investigación, C/Dr. Moliner 50, Burjassot, 46100 Valencia Spain; 3 CEA, DAM, DIF, F-91297 Arpajon, France; 4 Theoretical Divisions, Los Alamos National Laboratory, Los Alamos, New Mexico 87545 USA

**Keywords:** Materials science, Phase transitions and critical phenomena

## Abstract

In this work, the melting line of platinum has been characterized both experimentally, using synchrotron X-ray diffraction in laser-heated diamond-anvil cells, and theoretically, using *ab initio* simulations. In the investigated pressure and temperature range (pressure between 10 GPa and 110 GPa and temperature between 300 K and 4800 K), only the face-centered cubic phase of platinum has been observed. The melting points obtained with the two techniques are in good agreement. Furthermore, the obtained results agree and considerably extend the melting line previously obtained in large-volume devices and in one laser-heated diamond-anvil cells experiment, in which the speckle method was used as melting detection technique. The divergence between previous laser-heating experiments is resolved in favor of those experiments reporting the higher melting slope.

## Introduction

The 5d transition metals have always attracted considerable interest in the scientific community. This is due to their outstanding mechanical and thermal properties, making them extremely important in the field of fundamental physics as well as for the development of new high pressure technologies. In particular, due to their high yield strength, high ductility and phase stability, metals like rhenium (Re) and tungsten (W) are often used as gasket material for static experiments at extreme conditions. Whereas, noble metals such as platinum (Pt) and gold (Au) are generally used as pressure standard both in static (large volume press and diamond anvil cell (DAC)) and dynamic (shock wave (SW) and ramp compression) experiments. This is due to the high stability of their solid phase over a wide range of pressure (*P*) and temperature (*T*) (up to several Mbars and thousands of K) and their chemical inertness.

Among the 5d noble metals, Pt has a face-centered cubic (fcc) phase and, due to its good coupling with infrared lasers, it is often used as a laser absorber in laser-heating experiments. Due to its use as standard material in both static and dynamic experiments, several theoretical and experimental studies have been performed to characterize its thermal equation of state (EoS) in a *P-T* range between ambient and 660 GPa and 3200 K, respectively^1–9^.

**Figure 1:**
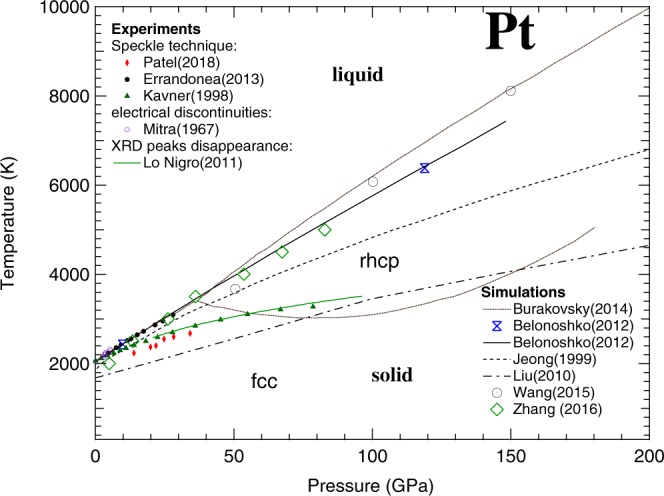
State of the art of the phase diagram of Pt as experimentally measured (solid symbols) and theoretically calculated (lines and empty symbols). The dotted lines represent the solid-solid (fcc to rhcp) and solid-liquid phase boundaries predicted in Burakovsky *et al*.^23^. In this figure all the studies described in the text are reported with the only exception of the one by Strong *et al*.^10^ (results similar to Mitra *et al*.^11^) for clarity reasons.

In DAC melting experiments, Pt can be heated up to extreme *T* at high *P*. Furthermore, providing the temperature of the solid-liquid transition in SW experiment is important to reduce the Hugoniot to room temperature. For these reasons, several experimental studies have been performed to characterize the melting *T* of Pt at high *P*^10–14^. Early Pt melting experiments^10,11^ were performed below 10 GPa and are in reasonable agreement with each other. These measurements were performed in large-volume devices. The sample, composed of a Pt wire (also used as thermocouple for temperature measurement), was heated up by an AC current and the melting was determined from the discontinuity in the measured resistance of the sample. Later experiments, performed in laser-heated (LH) DAC using Pt foil as a sample and MgO as insulating material, have extended the investigated range up to 80 GPa^12–15^. In these studies, the temperature was measured by spectral radiometry i.e. by fitting the radiated signal from the sample surface with a Planck function using the grey-body approximation^16^. The visual observation of the sample was used to detect the melting, both by direct observation of “flickering” in the laser hot-spot^12^ or, by the observation of movement on the sample surface via speckle technique^13,14^. However, with the notable exception of the study of Errandonea^13^, the melting curves obtained by these techniques^12,14^ are considerably lower than the one extrapolated from Mitra *et al*.^11^ using the Lindeman^17^ criterion, with a difference of almost 1600 K at 80 GPa. Results similar to the one obtained in Kavner *et al*.^12^ were also obtained in a LH-DAC experiment using the disappearance of diffraction lines as melting criterion^15^ (see Fig. 1).

Several theoretical calculations have also been performed to predict the melting line of Pt, with conflicting results. In particular, a molecular dynamic (MD) simulation performed by Zhang *et al*.^18^ agrees with the results obtained in large volume press^11^, whereas other MD simulations by Jeong *et al*.^19^ and Liu *et al*.^20^ predict melting lines of Pt that, at 100 GPa, are 850 K and 2200 K lower than the one extrapolated from the results of Mitra *et al*.^11^, respectively (see Fig. 1). Another MD simulation by Wang *et al*.^21^ predicts a steeper melting line, agreeing with Jeong *et al*.^19^ data at 50 GPa. However, above 100 GPa, the curve shows melting temperatures higher than the ones reported by Zhang *et al*.^18^. Recent *ab initio* simulations performed using the Z method^22,23^, seemed to agree with the results obtained by Mitra *et al*.^11^ and Errandonea^13^. However, a solid-solid phase transition (from the fcc to a randomly disordered hexagonal close-packed (rhcp) phase) predicted for Pt by Burakovsky *et al*.^23^ using the inverse Z method would place a limit on its application as standard material (see Fig. 1). Both the “direct” and inverse Z methods, constituting the Z methodology, are described in detail elsewhere^23^.

In Fig. 1, the theoretical and experimental results reported so far are summarized. The discrepancy of about 4000 K observed between the highest and the lowest reported melting curves at 150 GPa is mainly due to the difficulty in performing experiments and simulations at these extreme *P-T* conditions. However, such an uncertainty in the melting determination is unacceptable for a standard material, especially considering the recent advances in DAC technologies. The multi-Mbar domain is now becoming experimentally accessible^24,25^. This advance in high-pressure techniques requires standards which are reliable at multi-Mbar pressure range and temperature of thousands of Kelvins.

LH-DAC technology, combined with synchrotron X-ray diffraction (XRD) has now become a challenging but routinely used technique for the *in situ* characterization of phase diagram and melting line of samples at *P* up to 3 Mbar and *T* of the order of 5000 K^26^. In particular, it allows “time resolved” analysis of the structural, chemical and textural evolution of the sample to be performed as a function of *P* and *T*. In addition, the nature of the XRD technique also provides an objective melting criterion, i.e. the appearance of a diffuse halo in the diffraction pattern due to the scattering from the liquid sample. Thanks to the advance in this technique (metrology included) it was recently possible to shed light on several discrepancies observed on other metals melting curves (e.g. Pb^27^, Ta^28^, Fe^29^, Ni^30^ and Mo^31,32^).

For these reasons, we have decided to perform a new characterization of the phase diagram and melting curve of Pt both experimentally, using LH-DAC combined with synchrotron XRD technique, and theoretically, using *ab initio* calculation based on the Z method. The study is aimed not only to solve previous discrepancies but also to extend the pressure-temperature range covered by previous experiments.

## Results

### Experiments

Several experimental runs have been performed both at the Diamond Light Source (DLS), at the European Synchrotron Radiation Facility (ESRF) and at Soleil. The experimental conditions for all the experiments are reported in the Methods section. During the experiments, it was possible to cover a *P-T* range between 10 GPa and 110 GPa and between ambient temperature and 4800 K, respectively. In the investigated *P-T* range, only the fcc phase of Pt, the B2 phase of KCl and the B1 phase of MgO were observed. No structural distortions of the fcc phase of Pt or any chemical reactions^33^ were detected.

**Figure 2:**
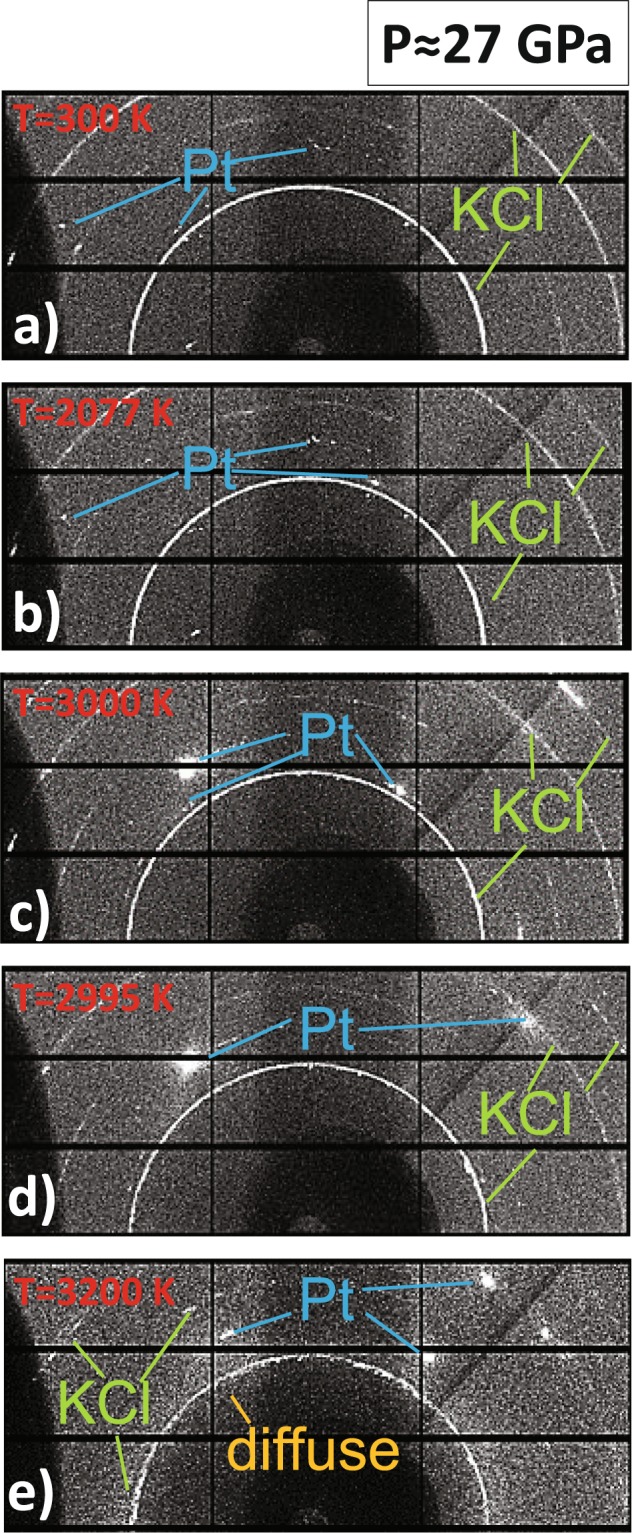
Textural evolution of a Pt foil embedded in KCl pressure transmitted medium measured at (**a**) ambient temperature, (**b**) 2077 K, (c) 3000 K, (**d**) 2995 K and (**e**) 3200 K.

During each experimental run it was possible to study the textural evolution of the sample (and the insulating material) with the increasing temperature. In Fig. 2 the textural evolution observed in a run at ~27 GPa is reported from ambient temperature (after laser alignment) up to 3200 K.

The signal from Pt evolves from a well oriented grain-like signal at ambient temperature, into a single crystal-like one at around 3000 K. The single crystal spots present sign of fast-recrystallization as, at similar temperatures and after only few seconds (Fig. 2(c,d)), diffraction spots generating from the same lattice planes appear at different azimuthal angles. Finally, with the temperature increase, the appearance of a diffuse halo, corresponding to liquid Pt, is clearly visible on the diffraction pattern (Fig. 2(e)). It is also possible to observe an axial temperature gradient in the sample (confirmed by the measured temperature from both sides) characterized by the combined presence of solid single crystal spots and diffuse signal from liquid sample.

In Fig. 2, the texture of KCl remains similar for almost the entire investigated *T* range. The only exception is the signal at 3200 K (Fig. 2(e)) where the KCl starts showing larger grains.

A critical examination of the temperature measurement has been performed for each laser-heating ramp. Temperatures were determined by fitting the radiated thermal signal with a Planck function in the grey-body approximation (as explained in the Methods section). Wien and two-colors pyrometry were used to determine the error in each temperature measurement following the procedure described in Benedetti & Loubeyre^34^. During the temperature ramps performed at DLS, the temperature was measured from both sides of the sample at the same time. The experimental temperature was considered as the average between these two measurements. The error was taken as the maximum value between the difference in the measured temperatures and, the full-width at half maximum (FWHM) in the histogram of the two-colors pyrometry^34^. Whereas, during the ramps performed at the ESRF, the temperature was measured only from the upstream side of the sample and the error was taken as the FWHM of the histogram of the two-colors pyrometry.

The reliability of the *T* measurement affects the error in the *P* measurement. The pressure is measured from the EoS of Dewaele *et al*.^35^ under the assumption that the KCl and the Pt are at the same temperature. However, the insulating material is placed between the sample (at *T* of thousands of K) and the diamonds (close to ambient *T*) and experience big thermal gradients. Assuming a temperature of KCl at the diamond interface of 300 K, it is possible to estimate the maximum error in pressure given by the EoS of Dewaele *et al*.^35^. Here, it varies between 2 GPa at 2000 K and 5 GPa at the maximum *T* reached during the experiment.

**Figure 3:**
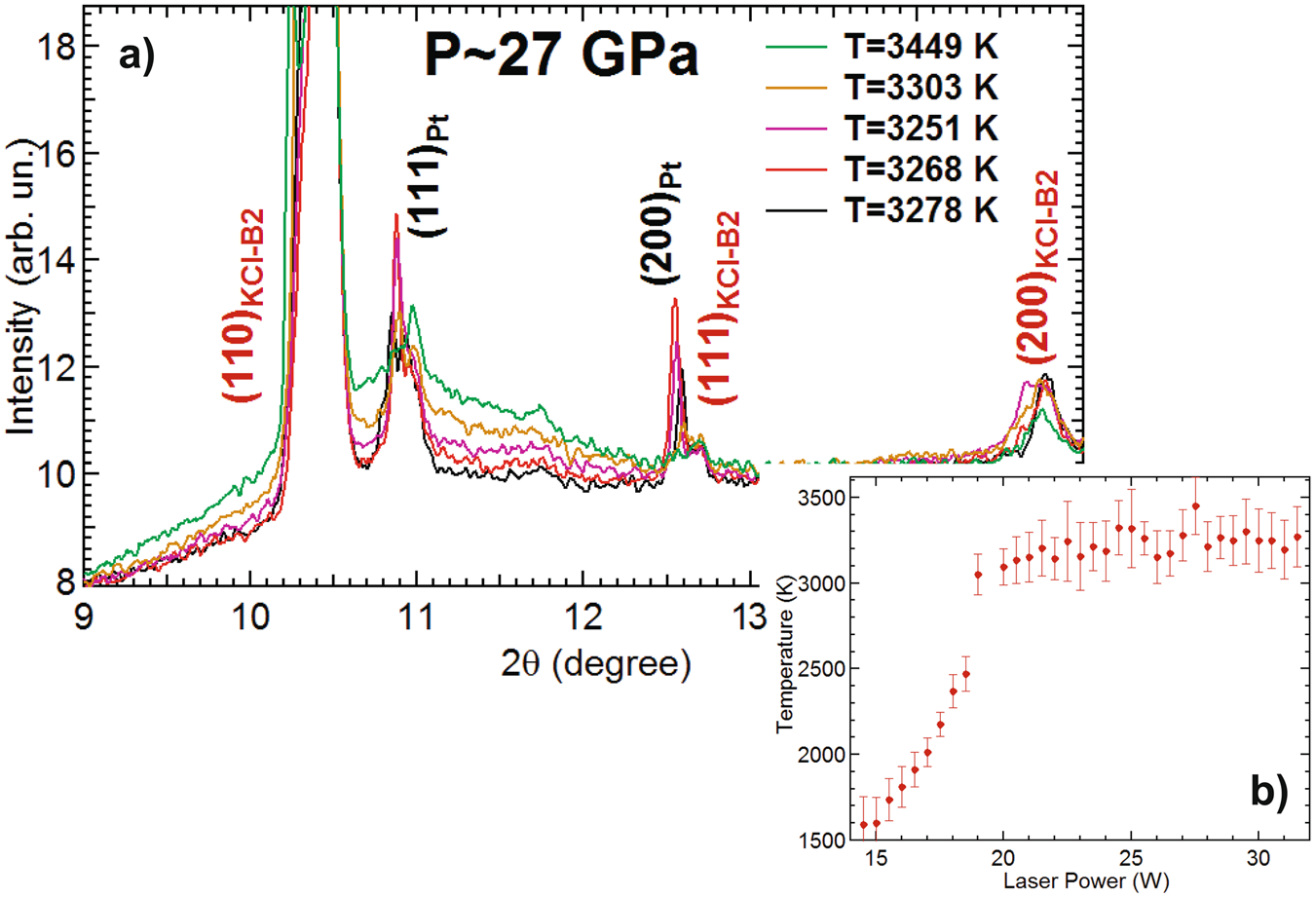
(**a**) Integrated diffraction signal of Pt foil at 27 GPa at five different temperatures, before and after the onset of the solid-liquid phase transition. (**b**) Corresponding temperature plateau in the temperature vs laser power plot.

During three of the experimental runs (at 26 GPa, 27 GPa and 50 GPa) a clear signal of diffuse was observed in the XRD pattern. An example is reported in Fig. 3a showing a selection of XRD patterns at different temperatures.

In all of the three cases, the appearance of a plateau in the temperature vs power (often used as melting criterion^28^) was observed (see Fig. 3b).

In Fig. 3a, the evolution of the diffraction signal at the onset of the melting is reported for the ramp performed at 27 GPa. The solid signal at 3278 K (in black) start showing a clear signal of diffuse at 3303 K (in platinum). The signal increases with increasing temperature as a large part of the sample starts melting. The observation of this behavior underlines the strength of the XRD technique for *in situ* characterization of melt in LH-DAC experiments. However, there is a high possibility of overestimating the melting temperature when the molten zone at the sample surface is too thin to create a measurable XRD signal. In a paper focused on the characterization of the melting line of titanium, Stutzmann *et al*.^36^ proposed a method to minimize the possibility of overestimating the melting temperature in XRD experiments performed in LH-DAC i.e. by measuring the fluctuations in the background signal around the diffraction peak closest to the diffuse signal. In the example reported in Fig. 3, the background signal around the 111 peak of Pt at 11.2 degree, shows a first increase in the fluctuations between 3251 K and 3268 K, providing an onset temperature for the solid-liquid transition of Pt at 27 GPa of (3260 ± 150) K. In this case, the value obtained using the method proposed in Stutzmann *et al*.^36^ agrees with the one obtained by averaging the temperature values at the plateau in the temperature vs laser power plot (Fig. 3b) i.e. (3200 ± 150) K.

Temperature plateaus were also observed in other runs in KCl where the diffuse signal of melting was not detected. In all the above mentioned runs, the sample presented a hole after quenching. The absence of a diffuse XRD signal can be caused by a misalignment of the temperature reading optics with respect to the X-rays due to thermal effects, or by the molten Pt moving away from the hot spot, as attested by the presence of a hole on the quenched sample.

Additional runs using MgO as pressure transmitting medium were performed to confirm the melting line using a different sample geometry. However, these runs were unsuccessful as for many trials, the laser made a hole in the sample below 2500 K at 70 GPa. Diffusion of the solid sample through the transmitting medium caused by Soret effect has been previously reported for other metals^28^. Probably, the numerous grain boundaries in MgO powder facilitate such a diffusion. KCl pellets appear here as a more effective sample container, with a diffusion happening only after melting.

The good agreement between the melting temperatures measured with XRD and the temperature plateaus suggest that the appearance of a plateau in the temperature vs laser power plot is a reliable melting criterion for Pt when KCl is used as pressure transmitting medium. For these reasons, additional off-line experiments were performed on Pt in KCl using the temperature plateau as melting criterion. In these runs, the samples were prepared as for the experiments at DLS. The temperature was measured by spectral-radiometry and the pressure by the ruby fluorescence method at 300 K^3^. The melting temperature obtained using this method are (4700 ± 300) K and (5000 ± 300) K at 70 GPa and 80 GPa, respectively. The thermal pressure was estimated from the results obtained during the on-line runs.

**Figure 4:**
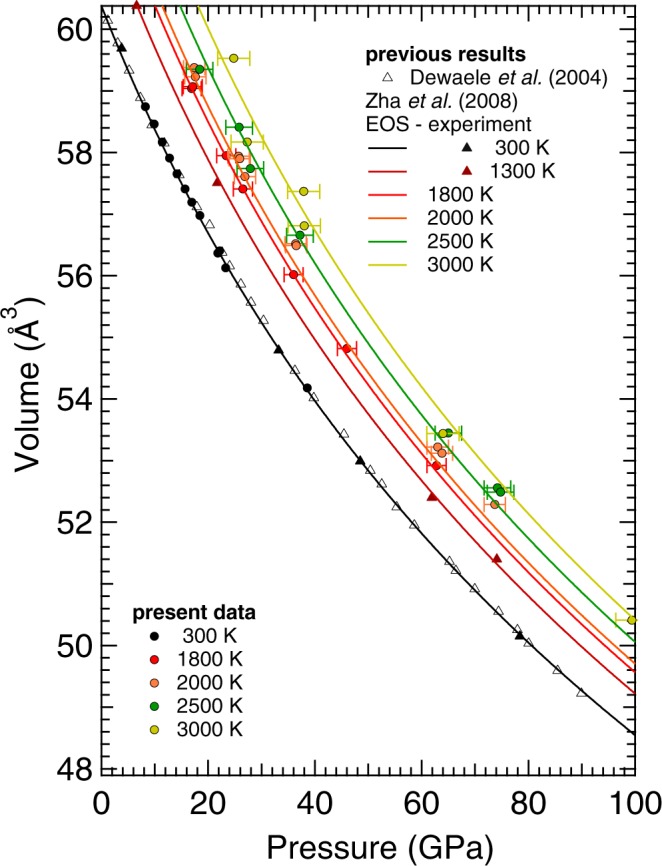
Thermal EoS of Pt (solid lines) as reported in Zha *et al*.^7^. Compared with the experimental data (solid symbols) obtained in the present study and in Dewaele *et al*.^1^. The temperature colour code is the same for all the reported studies to ease the lecture to the reader.

Several theoretical models have been used to predict the thermal expansion of Pt in the literature^4–8^. In Fig. 4 the model proposed by Zha *et al*.^7^ is compared with the experimental data obtained in the present study (using KCl as pressure medium) and in Dewaele *et al*.^1^ at different temperatures. The current data have been obtained using KCl as X-ray pressure gauge^35^, assuming that the temperature of KCl is identical to the temperature measured at the surface of the platinum sample. The thermal gradients within the KCl, even if they reach 1000 K, produce a relatively low pressure uncertainty of 2 GPa because of the low thermal pressure in this medium^35^. This uncertainty can explain the scatter as a function of temperature of the present experimental data, as seen in Fig. 4. Therefore, group of temperatures were defined in a range of ±150 K. In particular, data at 300 K, 1800 K, 2000 K, 2500 K and 3000 K were used in Fig. 4.

The thermal EoS described in Zha *et al*.^7^ was determined extrapolating the internal resistive heating DAC data, obtained up to 80 GPa and 1900 K, with both thermodynamic and Mie-Grünesein-Debye methods.

The comparison between the present results and the thermal EoS of Zha *et al*.^7^ shows a good agreement for the data obtained at 300 K and 1800 K. At 2000 K and 2500 K, the results agree within the experimental error. However, it is possible to observe a divergence in the results with the increasing pressure. At 74 GPa, the present volumes are 0.84% and 0.64% larger than the one predicted by the thermal EoS of Zha *et al*.^7^ at 2000 K and 2500 K, respectively.

A different situation is observed for the data at 3000 K. Even though the present results agree within the experimental error with the thermal EoS of Zha *et al*.^7^ the data seem to oscillate around the value predicted by the thermal EoS. It is important to underline the fact that the thermal EoS of Zha *et al*.^7^ was established from data collected up to 1900 K. Therefore, the behavior observed at higher *T* could be an indication of anharmonic effects not considered in the model. However, it cannot be ignored the possibility for the volume to have been measured from a region colder than those used to detect the temperature, due to small misalignment between the temperature reading diagnostic and the x-rays or, due to thermal gradients developed within the sample. Future studies should be carried out to clarify this issue.

## Calculations

**Figure 5:**
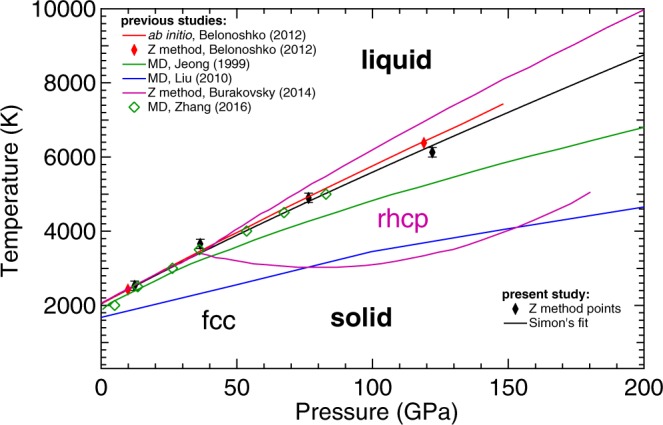
Results obtained in the present study from *ab initio* calculations using the Z-method (black points and line) compared with previously theoretical calculation using Z-method^22,23^ and MD simulations^18–20^.

In Fig. 5 the *ab initio* results obtained in the present study using the Z-method (black points) are reported together with previous theoretical calculations. The *P-T* coordinates of the obtained melting points are: (12.3 GPa, 2350 K), (36.5 GPa, 3660 K), (76.4 GPa, 4900 K) and (122 GPa, 6130 K). The present results have been fitted with a Simon’s equation (Eq. 1), being the temperature in K and pressure in GPa:

1 $${\rm{T}}({\rm{P}})=2041.7{(1+P/44.0)}^{0.85}$$

The obtained Simon equation is reported as a black continuous line in Fig. 5.

The results of the present calculation are in good agreement with the one previously obtained by Belonoshko *et al*.^22^ (red line in Fig. 5) using a similar method but considering a supercell with a smaller number of atoms (108 Pt atoms instead of the 500 atoms used here) and estimating the melting line using only two melting points and a Simon’s equation. They also agree with the more recent results of Burakovsky *et al*.^23^ (purple line in Fig. 5) in a pressure range between ambient and 50 GPa. However, in that work for pressure higher than 50 GPa, at high *T* a different solid phase (rhcp) is predicted to be more stable than the fcc. As a consequence, the two simulated melting curves start diverging. The calculations performed considering melting from the fcc phase give lower values than the melting curve calculated from the rhcp phase. In case of a solid-solid phase transition, a change in the melting slope should be detected (the melting curve should have a kink). This is not observed in the present experiments.

Concerning the results obtained by MD simulation, both calculations, Jeong *et al*.^19^ (in green) and Liu *et al*.^20^ (in blue) results lower than the present study by 2000 K and 4000 K at 200 GPa, respectively. In the case of Jeong *et al*.^19^ the lower melting temperature obtained could be related to the use of a different parametrization of the electron-core interaction and the use of a smaller period of time steps to stabilize the system. In the case of Liu *et al*.^20^, melting was determined using solid–liquid coexistence approach and a different computing code, which clearly leads to a large underestimation of the melting temperature not only at HP, but also at ambient pressure (see Fig. 5). Indeed, MD calculations carried out using a tight-binding (i.e. not *ab initio*) approximations^18^ give a melting curve more similar to the one here determined than the simulations of Jeong and Liu.

## Discussion

**Figure 6:**
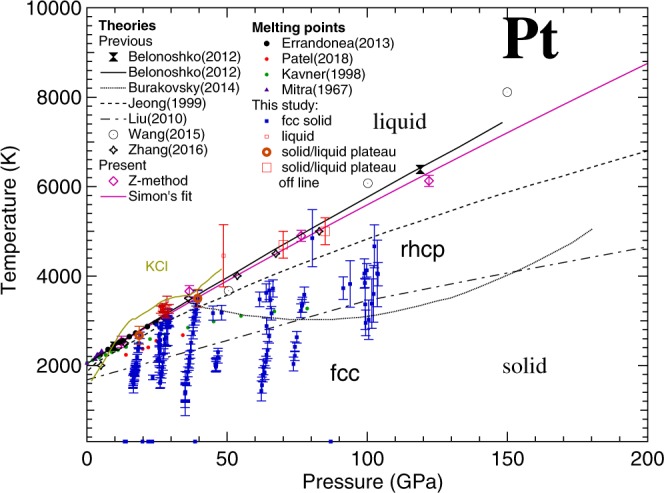
Phase diagram of Pt as measured (blue and red squares and orange circles) and simulated (purple point and solid line) in the present study, compared to previous results. The KCl melting curve obtained in Boehler *et al*.^37^ is reported as information.

In Fig. 6, the results obtained in the present study are reported together with previous theoretical and experimental data. In the graph, the melting line of KCl^37^ has also been plotted to confirm the reliability of the collected data. Eight runs have been performed using XRD in a *P-T* range between 10 GPa and 110 GPa and ambient temperature and 4800 K, respectively. In three runs at 26 GPa, 27 GPa and 50 GPa, respectively, a clear onset of melting was visible. During all runs, only the fcc phase of solid Pt was measured. The temperature of the plateaus in T vs laser power ramps measured online or offline are also reported. All these points are consistent, within experimental error bars, with the Simon fit of the melting points calculated with the Z method (Eq. (1)).

The obtained melting points obtained here are in agreement with the experimental results of Mitra *et al*.^11^ (up to 5 GPa) and Errandonea^13^ (up to 30 GPa). The theoretical calculations of Belonoshko *et al*.^21^ and Zhang *et al*.^17^ when melting is assumed to take place directly from the fcc phase, also produced a similar melting curve.

Concerning the solid part of the phase diagram of Pt, during the present study, solid fcc Pt has been observed at temperature higher than the melting line experimentally measured by Kavner *et al*.^12^ and Patel *et al*.^14^ and calculated in the MD simulation of Liu *et al*.^20^ and Jeong *et al*.^19^ In addition, we have explored the proposed region of stability of the rhcp phase up to 110 GPa, not finding any evidence of a phase transition from fcc to rhcp or any other solid-solid phase transition.

The occurrence of the rhcp phase in Pt was suggested based on the inverse Z solidification simulations that brought up different close-packed polytypes as the final solid states^23^. The melting curve of the rhcp phase was tentatively associated with that of the 9 R phase (also known as Sm-type) as being the highest among those polytypes, i.e. the most-stable phase at temperatures close to melting according to numerical simulations. However, several close-packed phases are in agreement with each other within the uncertainties of the inverse Z method itself. This suggest that the actual layer stacking could be non-periodic and, in principle, random. Likewise, any specific close-packed polytype can emerge and remain the only one over a given *P-T* region. This includes also fcc, which can be easily transformed into rhcp by introducing a local disorder in the staking sequence of close-packed fcc. A good example to illustrate this phenomenon is gold, for which the lowest-energy polytype is predicted to be double hcp (dhcp), but only hcp (a very similar structure but with a different staking sequence of layers) is observed in experiment**s**^38^. It is therefore quite possible that in the *P-T* region in which rhcp-Pt is predicted to occur, in our experiments fcc remains the most stable close-packed polytype, such that fcc is the only solid structure of Pt observed in the whole *P-T* region that our experiments span.

The disagreement observed with the results of Kavner *et al*.^12^ can be explained by the nature of the adopted melting diagnostic. In fact, with respect to the present *in situ* characterization, the method used in Kavner *et al*.^12^ is only based on the interpretation of the “flickering” observed in the hot-spot on the sample surface, which has larger uncertainties than our measurements. Indeed, in the work by Kavner *et al*.^12^ at a given pressure there is a melting temperature scattering of several hundred degrees. The melting curve assumed by these authors corresponds to the lowest melting temperature observed at each pressure. Thus, it should be considered as a lower bound to the melting curve. In fact, if the highest melting temperatures reported by Kavner *et al*.^12^ are taken as the melting temperature (e.g., 3100 K at 30 GPa), their results agree well with the present ones.

On the other hand, it is inexplicable the difference observed with the results obtained by Patel *et al*.^14^. In fact, the melting diagnostic adopted in their study is the same one used by Errandonea^13^ i.e. speckle technique, whose results are confirmed by our study. With this method, a visible laser is used to create interference patterns on the sample surface. The movement on these patterns has been associated to the formation of melt. This method has recently been questioned as it cannot provide any *in situ* information about the physical properties of the sample^26–29^. In particular, it is impossible to discriminate movements caused by the appearance of melting from those caused by any solid-solid phase transition or chemical reactions of the sample. However, the speckle technique seems to provide reliable results for low reactivity metals such as Al^39^ and Cu^40^ and a similar situation should be expected for Pt in a moderate *P-T* range. The observed discrepancy could be attributed to some difference in the adopted metrology. However, the paper of Patel *et al*.^14^ does not provide enough information allowing a critical comparison with the results of Errandonea^13^ to be performed.

In conclusion, the results obtained in the present work shed light on the melting curve and phase diagram of Pt up to 110 GPa and 5000 K extending the *P-T* range covered by previous studies. The challenging experiments carried out allowed the determination of melting points and range of stability of the fcc phase in the investigated *P-T* region. The melting points experimentally obtained are pushing towards a solid-liquid boundary in agreement with the one extrapolated from Mitra *et al*.^11^ and Errandonea^13^ and in excellent agreement with theoretical calculations obtained with the Z method and *ab initio* description of electronic interactions. Furthermore, the experiments do not confirm the predicted existence of a solid-solid phase transition in the investigated *P-T* range.

## Methods

### Experimental

Several membrane DACs with culet sizes ranging from 300 µm to 100 µm where equipped with pre-indented and sparkle-erosion drilled Re gaskets. Pt samples were cut from ~2 µm thick foil. The obtained samples were squeezed between two FIB-cut KCl disks and loaded in the DAC’s high-pressure chambers. The KCl disks, acting as insulating material (both thermally and chemically) as well as pressure gauges, were oven dried at 200 °C for two hours before being loaded in the DAC. The experiments were performed at the extreme condition beamlines I15 and ID27 at the DLS and the ESRF, respectively. Two additional runs, made using oven-dried MgO as pressure transmitting medium, have been performed at synchrotron Soleil.

The polychromatic beam of the two beamlines was tuned to 29.5 keV and 33 keV, respectively for I15 and ID27. A Pilatus 2 M detector was used on I15, whereas ID27 was equipped with a MAR CCD detector. Both detectors were used to ensure fast data collections with a good signal/noise ratio. In both cases, the sample-to-detector distance was measured following standard procedure from the diffraction rings of a reference sample.

The loaded DACs were mounted on the two beamlines LH-systems^41,42^. Before each heating run, the sample was brought to the target pressure. The pressure was measured from the compression curve of the KCl according to the thermal equation of state (EoS) of Dewaele *et al*.^35^. Two 100 W Nd:YAG fibre lasers were individually focused on both sample surfaces. During the experiment, the temperatures of the sample were measured by spectral radiometry (between 450 nm and 950 nm) following the procedure described in Benedetti & Loubeyre^34^. The temperature was collected from both side of the sample on I15 and only on the upstream side on ID27 (according to the adopted experimental set-up). In order to prevent the X-ray beam sampling a radial *T* gradient on the sample surface, the two lasers were defocused to maximize the size of the hot spot at uniform *T*. The two lasers were coupled together and their relative positions were tuned to obtain a uniform hot spot over about 40 µm in diameter. Before and after each heating run the relative alignment of the X-rays with the lasers and the temperature reading was checked following the procedure described in Anzellini *et al*.^41^.

On ID27, the runs were performed in “ramp mode” i.e. the lasers power was linearly increased up to a chosen value while XRD and temperature measurements were performed continuously every few seconds. On I15, instead, the heating runs were performed in “trigger mode” i.e. both lasers were set to a target power, after 0.3 s a diffraction pattern and a temperature measurement were collected simultaneously. Then, 0.3 s after the XRD collection, both lasers were turned off.

In both cases, the laser’s target powers were increased until a diffuse ring (characteristic of a liquid sample) was detected on the diffraction pattern or, a hole was laser-drilled in the sample.

Several heating-runs were performed for each sample. In order to avoid any chemical contamination, each run was performed on a different region of the sample. The quality of the selected region was checked by XRD before each heating ramp.

An accurate analysis of the diffraction patterns was performed in order to detect the appearance of the melting and, to obtain structural and textural information about the sample and the insulating material.

During the analysis procedure, masks were applied on a per-image basis using the DIOPTAS suite^43^. The images were azimuthally integrated and a Le Bail analysis was performed using the TOPAS suite^44^. The structural measurements were compared to the temperature one in order to obtain a detailed *in situ* and “time resolved” analysis of the sample evolution with *P* and *T*.

### Theoretical

In the present work, the melting curve of Pt up to ∼120 GPa was determined using the Z method^45^. This method consists in mapping out a set of Z-shaped isochores (at a set of different volumes) in the *P-T* plane, each having the upper vertex at maximum superheating and the lower vertex on the melting curve, thereby determining the melting point at the corresponding volume. This method was developed to calculate melting curves in the NVE ensemble using first-principles-based software. The method has since been applied to the study of a large number of melting curves of different materials and comparison with experimental data gives good results^27–29^.

The present calculations have been carried out using the quantum molecular dynamics (QMD) code VASP, which is based on the density-functional theory. In order to perform an accurate characterization of the melting line of Pt, five melting points were calculated for fcc-Pt, using both the most sophisticated theoretical methods and the most advanced resources available at the present. A generalized-gradient approximation with the Perdew-Burke-Ernzerhof exchange-correlation functional was implemented. Computer simulations were performed on the LANL clusters Pinto and Badger. For the simulations large supercells with 500-atoms (5 × 5 × 5) fcc-Pt were used to eliminate any possible size effect. Calculations were done at four different volumes corresponding to lattice constants of 4.05, 3.95, 3.85, and 3.75 Å. A single Γ point was used, for which convergence to 1 meV/atom with such a large supercell is already achieved. Eighteen valence electrons were used, with the electron core-valence representation [^46^Pd 4f^14^] 5 s^2^ 5p^6^ 5d^9^ 6s^1^. The valence electrons are represented with a plane-wave basis set with a cutoff energy of 370 eV, while the core electrons are represented by projector augmented-wave. The initial temperatures for melting simulations were chosen with increment of 250 K in each case, so that the error in the melting T from the method itself is ±125 K. The error in the melting *P* is negligibly small: around 0.5 GPa for the lowest *P* and 1–2 GPa for the other cases. The duration of each run was 15000–20000 time steps of 1 fs each.

## Data Availability

The datasets generated during and/or analysed during the current study are available from the corresponding author on reasonable request.

## References

[1] Dewaele A, Loubeyre P, Mezouar M (2004). Equation of state of six metals above 94 GPa. Phys. Rev. B.

[2] Holmes NC, Moriarty JA, Gathers GR, Nellis WJ (1989). The equation of state of platinum to 660 GPa (6.6 Mbar). J. App. Phys..

[3] Dorogokupets PI, Organov AR (2007). Ruby, metals, and MgO as alternative pressure scale: A semiempirical description of shock-wave, ultrasonic, x-ray, and thermochemical data at high temperature and pressure. Phys. Rev. B.

[4] Matsui M (2009). The temperature-pressure-volume equation of state of platinum. J. App. Phys..

[5] Sun T, Umemoto K, Wu Z, Jheng JC, Wentzcovitch RM (2008). Lattice dynamics and thermal equation of state of platinum. Phys. Rev. B.

[6] Huang X (2015). *In situ* synchrotron X-ray diffraction with laser-heated diamond anvil cells study of Pt up to 95 GPa and 3150 K. RSC Adv..

[7] Zha CS (2008). P-V-T equation of state of platinum to 80 GPa and 1900 K from internal resistive heating/x-ray diffraction measurements. J. App. Phys..

[8] Xiang S, Cai L, Bi Y, Jing F (2005). Thermal equation of state for Pt. Phys. Rev. B.

[9] Dorfman SM, Prakapenka VB, Meng Y, Duffy TS (2012). Intercomparison of pressure standards (Au, Pt, Mo, MgO, NaCl and Ne) to 2.5 Mbar. J. Geophys. Res..

[10] Strong HM, Bundy FP (1959). Fusion curves of four group VIII metals to 100000 atmospheres. Phys. Rev..

[11] Mitra NR, Decker DL, Vanfleet HB (1967). Melting curves of copper, silver, gold and platinum to 70 kbar. Phys. Rev..

[12] Kavner A, Jeanloz R (1998). High-presure melting curve of platinum. J. Appl. Phys..

[13] Errandonea D (2013). High-pressure melting curves of the transition metals Cu, Ni, Pd, and Pt. Phys. Rev. B.

[14] Patel NN, Sunder M (2018). High pressure melting curve of platinum up to 35 GPa. AIP Conf. Proc..

[15] Lo Nigro, G. Experimental investigation of the deep mantle melting properties. PhD thesis, University Blaise Pascal-Cermont-Ferrand II (2011).

[16] Walter MJ, Koga KT (2004). The effect of chromatic dispersion on temperature measurement in the laser-heated diamond anvil cell. Phys. Earth Planet. Inter..

[17] Lindemann, F. *The calculation of molecular vibration frequencies Z*. *Phys*, **11**, 609 (1910).

[18] Zhang B, Wang B, Liu Q (2016). Melting curves of Cu, Pt, Pd and Au under high pressure. Int. J. Mod. Phys. B.

[19] Jeong JW, Chang KJ (1999). Molecular-dynamics simulations for the shock Hugoniot meltings of Cu, Pd and Pt. J. Phys.: Condens. Matter.

[20] Liu ZL, Yang JH, Zhao ZG, Cai ZG, Jing FQ (2010). The anisotropy of shock-induced melting of Pt observed in molecular dynamics simulations. Phys. Lett. A.

[21] Wang PP, Shao JX, Cao QL (2016). Melting properties of Pt and its transport coefficients in liquid states under high pressure. Int. J. Mod. Phys. B.

[22] Belonoshko AB, Rosengren A (2012). High-pressure melting curve of platinum from ab initio Z method. Phys. Rev. B.

[23] Burakovsky L, Chen SP, Preston DL, Sheppard DG (2014). Z methodology for phase diagram studies: platinum and tantalum as examples. J. Phys.: Conf. Series.

[24] Dubrovinsky L, Dubrovinskaia N, Prakapenka V, Abakumov AM (2012). Implementation of micro-ball nanodiamond anvils for high-pressure studies above 6 Mbar. Nat. Comm..

[25] Dewaele A, Loubeyre P, Occelli F, Marie O, Mezouar M (2018). Toroidal diamond anvil cell for detailed measurements under extreme static pressure. Nat. Comm..

[26] Tateno S, Hirose K, Ohishi Y, Tatsumi Y (2010). The structure of iron in Earth’s inner core. Science.

[27] Dewaele A, Mezouar M, Guignot N, Loubeyre P (2007). Melting of lead under high pressure studied using second-scale time-resolved x-ray diffraction. Phys. Rev. B.

[28] Dewaele A, Mezouar M, Guignot N, Loubeyre P (2010). High melting points of tantalum in a laser-heated diamond anvil cell. Phys. Rev. Let..

[29] Anzellini S, Dewaele A, Mezouar M, Loubeyre P, Morard G (2013). Melting of iron at Earth’s inner core boundary based on fast x-ray diffraction. Science.

[30] Lord OT (2014). The melting curve of Ni to 1 Mbar. Earth Planet. Sci. Lett..

[31] Hrubiak R, Meng Y, Shen G (2017). Microstructures define melting of molybdenum at high pressures. Nature Communications.

[32] Santamaría-Pérez D (2009). X-ray diffraction measurements of Mo melting to 119 GPa and the high pressure phase diagram. J. Chem. Phys..

[33] Benedetti R, Loubeyre P (2004). Temperature gradients, wavelength-dependent emissivity, and accuracy of high and very-high temperatures measured in the laser-heated diamond cell. High. Press. Res..

[34] Ono S, Kikegawa T, Ohishi Y (2005). A high-pressure and high-temperature synthesis of platinum carbide. Solid. State Comm..

[35] Dewaele A (2012). High-pressure-high-temperature equation of state of KCl and KBr. Phys. Rev. B.

[36] Stutzmann V, Dewaele A, Bouchet J, Bottin F, Mezouar M (2015). High-pressure melting curve of titanium. Phys. Rev. B.

[37] Boehler R, Ross M, Boercker DB (1996). High-pressure melting curves of alkali halides. Phys. Rev. B.

[38] Dubrovinsky L (2007). Noblest of all metals is structurally unstable at high pressure. Phys. Rev. Lett..

[39] Alfe D, Vocadlo L, Price G, Gillan M (2004). Melting curve of materials: theory versus experiments. J. Phys.: Condens. Matter.

[40] Japel S, Schwager B, Boehler R, Ross M (2005). Melting of copper and nickel at high pressure: the role of d electrons. Phys. Rev. Lett..

[41] Anzellini, S. *et al*. Laser-heating system for high-pressure x-ray diffraction at the extreme condition beamline I15 at diamond light source. *J*. *Synch*. *Rad*. **25** (2018). 10.1107/S1600577518013383 PMC6225745 30407199

[42] PetigGirard S, Salamat A, Beck P, Weck G, Bouvier P (2014). Strategies for *in situ* laser heating in the diamond anvil cell at an X-ray diffraction beamline. J. Synch. Rad..

[43] Prescher C, Prakapenka V (2015). DIOPTAS: a program for reduction of two-dimensional x-ray diffraction data and data exploration. High Press. Res..

[44] Coelho AA (2018). TOPAS and TOPAS-Academic: an optimization program integrating computer algebra and crystallographic object written in C++. J. Appl. Cryst..

[45] Burakovsky L, Burakovsky N, Preston DL (2015). Ab initio melting curve of osmium. Phys. Rev. B.

